# Animal models are essential to biological research: issues and perspectives

**DOI:** 10.4155/fso.15.63

**Published:** 2015-11-01

**Authors:** Françoise Barré-Sinoussi, Xavier Montagutelli

**Affiliations:** 1 INSERM & Unité de Régulation des Infections Rétrovirales, Institut Pasteur, 75724 Paris, France; 2 Animalerie Centrale, Institut Pasteur, 75724 Paris, France

**Keywords:** animal models, animal protection, animal research, animal welfare, human diseases, preclinical studies

The use of animals for scientific purposes is both a longstanding practice in biological research and medicine, and a frequent matter of debate in our societies. The remarkable anatomical and physiological similarities between humans and animals, particularly mammals, have prompted researchers to investigate a large range of mechanisms and assess novel therapies in animal models before applying their discoveries to humans. However, not all results obtained on animals can be directly translated to humans, and this observation is emphasized by those who refute any value to animal research. At the same time, the place of the animals in our modern societies is often debated, particularly the right to use animals to benefit human purposes, with the possibility that animals are harmed. These two aspects are often mixed in confusing arguments, which does not help citizens and politicians to get a clear picture of the issues. This has been the case in particular during the evaluation of the European Citizen Initiative (ECI) ‘Stop Vivisection’ recently presented to the European Commission [1].

Humans and other mammals are very complex organisms in which organs achieve distinct physiological functions in a highly integrated and regulated fashion. Relationships involve a complex network of hormones, circulating factors and cells and cross-talk between cells in all the compartments. Biologists interrogate organisms at multiple levels: molecules, cells, organs and physiological functions, in healthy or diseased conditions. All levels of investigations are required to get a full description and understanding of the mechanisms. The first two, and in some instances three, levels of organization can be studied using *in vitro* approaches (e.g., cell culture). These techniques have become very sophisticated to mimic the 3D and complex structures of tissues. They represent major scientific advances and they have replaced the use of animals. On the other hand, the exploration of physiological functions and systemic interactions between organs requires a whole organism. It is, for example, the case for most hormonal regulations, for the dissemination of microorganisms during infectious diseases or for the influence of the intestinal microorganisms on immune defense or on the development of brain functions. In these many cases, no *in vitro* model is currently available to fully recapitulate these interactions, and investigations on humans and animals are still necessary. Hypotheses and models can emerge from *in vitro* studies but they must be tested and validated in a whole organism, otherwise they remain speculative. Scientists are very far from being able to predict the functioning of a complex organism from the study of separate cells, tissues and organs. Therefore, despite arguments put forward by the promotors of the ECI, studies on animals cannot be fully replaced by *in vitro* methods, and it is still a long way before they can.

Animal models have been used to address a variety of scientific questions, from basic science to the development and assessment of novel vaccines, or therapies. The use of animals is not only based on the vast commonalities in the biology of most mammals, but also on the fact that human diseases often affect other animal species. It is particularly the case for most infectious diseases but also for very common conditions such as Type I diabetes, hypertension, allergies, cancer, epilepsy, myopathies and so on. Not only are these diseases shared but the mechanisms are often also so similar that 90% of the veterinary drugs used to treat animals are identical or very similar to those used to treat humans. A number of major breakthroughs in basic science and medical research have been possible because of observations and testing on animal models. Most vaccines, which save millions of human and animal lives every year, have been successfully developed using animal models. The treatment of Type I diabetes by insulin was first established in the dog by Banting and McLeod who received the Nobel Prize in 1921 [2]. Cellular therapies for tissue regeneration using stem cells have been engineered and tested in animals [3]. Many surgical techniques have been designed and improved in various animal species before being applied to humans. The discoveries in which animal models played a critical role are indeed numerous and led to many Nobel Prizes.

It is, however, noticeable that the results obtained on animals are not necessarily confirmed in further human studies. Various reasons can be evoked. First, despite large similarities, there are differences between a given animal species and humans. For example, over 95% of the genes are homologous between mice and humans but there are also differences for example in the members of genes families, in gene redundancies and in the fine regulation of gene-expression level. These genetic differences translate into physiological differences which are increasingly better described and understood. While some people like the ECI promotors use these differences to refute the value of animal models, many including ourselves strongly advocate for further improving our knowledge and understanding of these differences and for taking them into account in experimental designs and interpretation of observations [4]. Moreover, these differences may provide opportunities to unravel novel mechanisms and imagine innovative therapies.

The second reason is due to genetic and physiological variations within each species or between closely related species. Laboratory mice have been developed as inbred strains which have highly homogeneous genetic composition to increase the reproducibility of results and the statistical power of experiments. Reports on animal models of human conditions often speak of ‘the mouse model of…’, referring in fact to observations made in a given genetic background. However, the clinical presentation often varies if another mouse strain is considered. A striking example is provided by a study published in November 2014 in *Science* by a team who reported that some mouse strains are fully resistant to Ebola virus, others die without specific symptoms and others develop fatal hemorrhagic fever [5]. Another example is the difference of responses to SIV, the monkey homolog to human HIV, between Rhesus macaques which develop simian AIDS and sooty mangabeys which do not develop symptoms despite high levels of circulating virus [6]. This range of responses reflects in fact the variety of clinical observations among human patients. These examples illustrate how animal models must be considered: no single animal model is able to mimic a given human disease which is itself polymorphic between patients, but the differences between strains or species provide unmatched opportunity to understand disease development and differential host response, and to eventually find new cures.

The second issue regarding the use of animals for scientific purposes is animal protection and welfare. This is the scope of the European Directive 2010/63/EU, which has set the regulatory framework for all animal research. Scientists have recognized for decades the importance of giving full consideration to three fundamental principles [7], which have become the backbone of the European Directive. First, animals must not be used whenever other, non-animal-based, experimental approaches are available, with similar relevance and reliability. Second, the number of animals used must be adjusted to the minimum needed to reach a conclusion. Third, all provisions must be taken throughout the procedures to minimize any harm inflicted to the animals. These principles, known as ‘the three Rs rules’, for replacement, reduction and refinement, have become the standard to which every project involving the use of animals is evaluated.

Animal research is conducted in compliance with regulatory provisions which cover the inspection and licensing of animal premises, the training and competence of all personal designing projects, performing animal procedures and taking care of animals and the mandatory authorization of every project by a competent authority upon ethical evaluation by an Animal Ethics Committee. The criteria for evaluation are based on the 3Rs rules and a cost–benefit analysis to evaluate if the potential harm to the animals, which must be reduced to the lowest possible level, is outweighed by significant progress in terms of knowledge on human or animal health. Regulation imposes that ethics committees include members concerned by animal protection and not involved in animal research. In response to the ECI, the European Commission has underlined, in a statement issued on 3 June 2015 [8], that animal experimentation remains important for improving human and animal health. At the same time, it is committed to promoting the development and validation of non-animal-based approaches, and to enforcing the application of the 3Rs rules by all players, including the research community. Europe has therefore implemented one of the strictest regulatory frameworks for the protection of animals used in research.

The greatest challenges faced by modern biomedical research concern complex, multifactorial, diseases such as cancer, cardiovascular diseases, infectious diseases, neurodegenerative disorders, pathological consequences of aging among others, for which all experimental approaches are indispensable because of their complementarity: biochemistry, genomics, cell culture, computer modeling, animal model and clinical studies. Research on relevant, carefully designed, well-characterized and controlled animal models will remain for a long time an essential step for fundamental discoveries, for testing hypotheses at the organism level and for the validation of human data. Animal models must be constantly improved to be more reliable and informative. Likewise, animal protection requires permanent consideration. These two objectives, far from being antagonistic, must be anchored in high-quality science.
